# Cosavirus, Salivirus and Bufavirus in Diarrheal Tunisian Infants

**DOI:** 10.1371/journal.pone.0162255

**Published:** 2016-09-15

**Authors:** Siwar Ayouni, Marie Estienney, Sabeur Hammami, Mohamed Neji Guediche, Pierre Pothier, Mahjoub Aouni, Gael Belliot, Alexis de Rougemont

**Affiliations:** 1 Centre National de Référence des virus entériques, Laboratoire de virologie-sérologie, Pôle Technique de Biologie, CHU de Dijon, Dijon, France; 2 Faculté de Pharmacie, Université de Monastir, Monastir, Tunisie; 3 Service de Pédiatrie, Hôpital Universitaire Fattouma-Bourguiba, Monastir, Tunisie; 4 Faculté de Médicine, Université de Monastir, Monastir, Tunisie; 5 UMR PAM A 02.102 Procédés Alimentaires et Microbiologiques, Université de Bourgogne Franche-Comté/AgroSup Dijon, Dijon, France; University of Michigan, USA, UNITED STATES

## Abstract

Three newly discovered viruses have been recently described in diarrheal patients: Cosavirus (CosV) and Salivirus (SalV), two picornaviruses, and Bufavirus (BuV), a parvovirus. The detection rate and the role of these viruses remain to be established in acute gastroenteritis (AGE) in diarrheal Tunisian infants. From October 2010 through March 2012, stool samples were collected from 203 children <5 years-old suffering from AGE and attending the Children’s Hospital in Monastir, Tunisia. All samples were screened for CosV, SalV and BuV as well as for norovirus (NoV) and group A rotavirus (RVA) by molecular biology. Positive samples for the three screened viruses were also tested for astrovirus, sapovirus, adenovirus, and Aichi virus, then genotyped when technically feasible. During the study period, 11 (5.4%) samples were positive for one of the three investigated viruses: 2 (1.0%) CosV-A10, 7 (3.5%) SalV-A1 and 2 (1.0%) BuV-1, whereas 71 (35.0%) children were infected with NoV and 50 (24.6%) with RVA. No mixed infections involving the three viruses were found, but multiple infections with up to 4 classic enteric viruses were found in all cases. Although these viruses are suspected to be responsible for AGE in children, our data showed that this association was uncertain since all infected children also presented infections with several enteric viruses, suggesting here potential water-borne transmission. Therefore, further studies with large cohorts of healthy and diarrheal children will be needed to evaluate their clinical role in AGE.

## Introduction

Diarrhea remains a frequent illness throughout the world and causes the death of almost 6 million children annually, especially in developing countries. Besides well-documented enteric viruses, the list of viral pathogens causing acute gastroenteritis (AGE) is continuously growing with the emergence of new viruses. No less than three new types of virus have been discovered in diarrheal patients these last years: Cosavirus and Salivirus, two new genera in the *Picornaviridae* family since 2013, and Bufavirus from the *Protoparvovirus* genus of the *Parvoviridae*.

Cosavirus (CosV) was first identified in 2008 from children suffering from acute flaccid paralysis but has been later associated with diarrhea. It has been detected in feces from both patients with gastroenteritis and healthy subjects **[1]**. CosV has a single-stranded RNA genome of around 7.6 Kb organized in a typical picornavirus genome and has a wide genetic diversity: not less than 6 species (noted A to F) have already been described, of which CosV-A includes 24 different genotypes, and CosV-D includes 5 different genotypes **[2]**.

Salivirus (SalV) was first identified in 2009 **[3, 4]**. SalV has a single-stranded RNA genome of around 7.1Kb organized in a typical picornavirus genome. Although this virus is related, but distinct, to the Kobuvirus genus, SalV forms a genus that presently includes a single genotype with 2 clusters **[5]**. Human salivirus (also formerly called klasseviruses) has been associated with diarrhea and detected in feces from both gastroenteritis patients and healthy subjects from all continents, as well as in sewage from Spain and Hong-Kong, suggesting a widespread geographic distribution **[4]**.

Bufavirus (BuV) was first discovered in 2012 in fecal samples from children suffering from diarrhea in Burkina Faso, from which it get its name **[6]**. Thereafter, BuV was detected in diarrheal stool samples of children from other continents **[7–10]**. BuV has a single-stranded DNA genome of around 4.9Kb, which encodes nonstructural protein 1 (NS1) and viral structural protein (VP2). Three genotypes (BuV1, 2 and 3) have been described so far **[11]**, but diversity within the capsid gene suggests the possibility of several other genotypes **[6]**.

Whether these viruses are etiologic agents of human gastroenteritis remains unclear, but knowledge about their distribution and genetic divergence in humans is mounting. In this context, the detection rate and the role of these new viruses in AGE in diarrheal infants remain to be established.

## Methods

From October 2010 through March 2012, stool samples were collected from 203 children <5 years-old suffering from AGE and attending the Fattouma-Bourguiba Children’s Hospital in Monastir, Tunisia. The children’s median age (MA) was 7.0 mo (ranging 0.5 to 60 mo), and the sex ratio was 1.29. The study and the data collection procedure were approved by the Ethics and Research Committee of the Fattouma-Bourguiba Public Hospital. Informed consents were obtained verbally from the parents of the study participants and consigned in their clinical records in accordance to the Tunisian good clinical practices and hospital clinical investigations guidelines. The samples were anonymized before processing.

For each stool sample, nucleic acids were extracted from 800 μl of 10% fecal suspension in PBS on a Nuclisens® EasyMAG system (bioMérieux, Marcy l’Etoile, France), according to the manufacturer’s instructions. RNA/DNA was eluted in a final volume of 110 μL. CosV and SalV were screened by nested RT-PCR using primer sets targeting the 5’UTR region **[1, 12]**; and BuV by nested PCR using primer sets targeting the NS1 region **[6]**. Virus characterization was performed using primer sets targeting various regions: capsid (VP1) and polymerase (3Dpol) for CosV **[1, 2]**; capsid (VP0), helicase (2Chel) and polymerase (3Dpol) for SalV **[4, 13, 14]**; and the capsid protein region (VP2) for BuV using the following designed primers: ARUB259: 5’- ATCTCTTTGTTAACCTTGCTAGAAAAAAAG -3’ and ARUB261: 5’- TTASWWTGTGTAGTTWGGCATDSMTC -3’ giving a PCR product of 1710 nt. Primer details are provided in **Table 1**. RT-PCRs were performed using the Qiagen OneStep RT-PCR kit (Qiagen GmbH, Hilden, Germany) and PCRs with Novagen KOD Hot Start polymerase kit (EMD Millipore, Darmstadt, Germany) on an Eppendorf MasterCycler PCR machine (Eppendorf AG, Hamburg, Germany), according to the manufacturer’s instructions.

**Table 1:** Oligonucleotides used for detection and genotyping of cosavirus, salivirus and bufavirus in this study.

Virus	Method	Target	Primer	Nucleotide Sequence	Reference
**Cosavirus**	RT-PCR	5’UTR	DKV-N5U-F1	CGT GCT TTA CAC GGT TTT TGA	Kapoor *et al*, 2008 **[1]**
	(1^st^ round)		DKV-N5U-R1	GGT ACC TTC AGG ACA TCT TTG G	
		VP1	INO-VP1-F1	GAI CAR GCI ATG ATG GGI AC	Kapusinszky *et al*, 2012 **[2]**
			INO-VP1-R2	GCI GGI CCI GGR TTK GWY TC	
		3Dpol	DKV-IF1	CTA CCA RAC ITT YCT IAA RGA	Kapoor *et al*, 2008 **[1]**
			DKV-IR1	GCA ACA ACI ATR TCR TCI CCR TA	
	PCR	5’UTR	DKV-N5U-F2	ACG GTT TTT GAA CCC CAC AC	Kapoor *et al*, 2008 **[1]**
	(2^nd^ round)		DKV-N5U-R2	GTC CTT TCG GAC AGG GCT TT	
		VP1	INO-VP1-F1-2	GCC ATG ATG GGI ACI TWY DCI ATI TGG GA	Kapusinszky *et al*, 2012 **[2]**
			INO-VP1-R3	TAR TCI GGR TAI CCR TCR AA	
		3Dpol	DKV-IF2	CTA CCA GAC ATT TCT CAA RGA YGA	Kapoor *et al*, 2008 **[1]**
			DKV-IR2	CCG TGC CAG AIG GIA RIC CIC C	
**Salivirus**	RT-PCR	5’UTR	SAL-L1	CCC TGC AAC CAT TAC GCT TA	Shan *et al*, 2010 **[12]**
	(1^st^ round)		SAL-R1	CAC ACC AAC CTT ACC CCA CC	
		VP0	LG0119	GCT AAC TCT AAT GCT GCC ACC	Holtz *et al*, 2009 **[4]**
			LG0136	GCT AGG TCA GTG GAA GGA TCA	
		2Chel	KL-2C-F1	CTC GCY GAG GAC ATC ACG GA	Han *et al*, 2010 **[13]**
			KL-2C-R1	GTA CAG GTA CAC RAC CAG TGG CT	
		3Dpol	LL-3D-F1	GAA GAT GCC ATT CGT GGT CTC	Li *et al*, 2009 **[14]**
			LL-3D-R1	AGT CCA GAA CAC GAC CAG GTT	
	PCR	5’UTR	SAL-L2	ATT GAG TGG TGC ATG TGT TG	Shan *et al*, 2010 **[12]**
	(2^nd^ round)		SAL-R2	ACA AGC CGG AAG ACG ACT AC	
		VP0	KLVPF	GTC ACY CCM AAC ACC TCC ACT GAA G	Han *et al*, 2010 **[13]**
			KLVPR	TTC TGC RCC ATC RGC TCC CGA	
		2Chel	KL-2C-F2	AAT CTG CTG CCC AGG CCG C	Han *et al*, 2010 **[13]**
			KL-2C-R2	AGG GAG ATG GCR GAG AGA GCT GT	
		3Dpol	LL-3D-F2	CTT TCC CAA TCT CCT GGC TAC	Li *et al*, 2009 **[14]**
			LL-3D-R2	GAA GGA CAG AGG GGA TAG TGG	
**Bufavirus**	RT-PCR	NS1	BF.F1	TCA ACA ATC ACT CAG GCA AAT GG	Phan *et al*, 2012 **[6]**
	(1^st^ round)		BF.R1	AGT TTG CCT GGA TGT TCT TTG A	
	PCR	NS1	BF.F2	CTA ACA CTG GTA CTT GCT ATG GAC	Phan *et al*, 2012 **[6]**
	(2^nd^ round)		BF.R2	TTC TCT GGT GAT GAT TCT TTT GTC	

Sequences were obtained with the ABI Big Dye sequencing kit on an ABI 3130XL sequencer (Applied Biosystems, Waltham, USA). Phylogenetic analysis were performed using using MEGA6 software **[15]**. After sequence alignment using the MUSCLE programme with a maximum of 64 iterations **[16]**, phylogenetic trees were inferred using the Maximum Likelihood method based on the Tamura-3-parameter model with a discrete gamma distribution, which was the best-fit DNA substitution model for the nucleotide dataset submitted. Bootstrap values were calculated from 1000 replicates. The nucleotide sequences were deposited in the GenBank database under the accession numbers: KU362760 to KU362792.

All samples were tested for norovirus (NoV) and group A rotavirus (RVA) by RT-qPCR. Positive samples for the three screened viruses were also tested for astrovirus (AstV) and sapovirus (SaV) by RT-qPCR, adenovirus (AdV) by qPCR and Aichi virus (AiV) by RT-PCR then genotyped when technically feasible, using the NRC’s PCR screening and typing procedures as reported in **S1 Table [17–30]**. Secretor status of positive individuals has been determined by genotyping on blood samples by using the methods described in our previous studies **[31, 32]**.

## Results and Discussion

**Fig 1:**
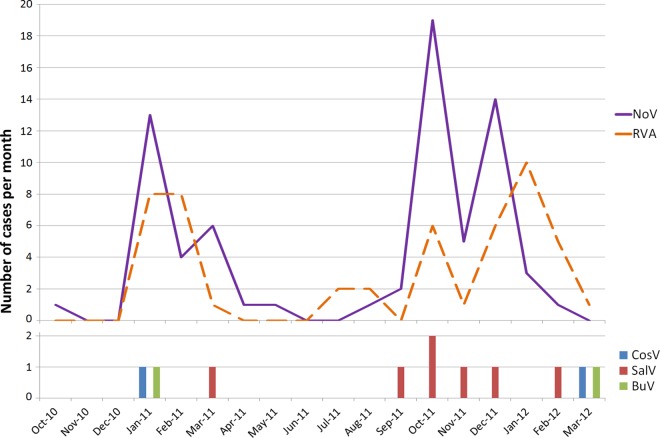
Temporal distribution of norovirus, rotavirus, cosavirus, salivirus and bufavirus in Tunisian children from October 2010 to March 2012.

During the study period, 71 (35.0%) children were infected with norovirus and 50 (24.6%) with rotavirus, of which 11 were mixed infections. In all, 11 (5.4%) samples were positive for one of the three investigated viruses: 2 (1.0%) CosV (MA = 12.0 mo.), 7 (3.5%) SalV (MA = 6.0 mo.) and 2 (1.0%) BuV (MA = 20.8 mo.). Of note, all individuals infected by these new viruses were secretors. No mixed infections involving these viruses were found, but multiple infections with 1 to 4 viruses responsible for GEA in humans were found in all cases **(Table 2)**. These mixtures of several enteric viruses, particularly with non-enteric adenoviruses, suggest here an environmental contamination from soiled waters. While both CosV and BuV infections occurred during winter in 2 different seasons, most SalV infections occurred in the autumn of the second season of the survey **(Fig 1)**. However, the number of tested stools being rather limited, a larger sampling will be required to confirm any seasonality pattern.

**Table 2:** Demographic characteristic and detected enteric viruses in patients with cosavirus, salivirus or bufavirus infections.

New viruses detected	Genotype	Sample ID	Age, mo / sex	Sample date	Other viruses tested*						GenBank Accession Numbers
					NoV	RVA	AstV	AdV	SaV	AiV	
**CosV**	A10	H036	9/M	2011/01/21	GII.g/GII.1	‒	‒	AdV-2	GI.2	AiV-A	KU362764^a^, KU362766^b^, KU362784^c^, KX721253^d^
	A10	H226	3/F	2012/03/15	‒	‒	‒	‒	**+**	‒	KU362765^a^,KU362785^c^KX721254^d^
**SalV**	A1	H010	27/M	2011/03/09	‒	G2P[4]	‒	‒	**+**	‒	KU362767^d^, KU362773^a^, KU362779^b^, KU362786^c^
	A1	H108	13/M	2011/12/03	‒	G9P[8]	‒	**+**	GII.1	‒	KU362768^d^, KU362774^a^, KU362780^b^, KU362787^c^
	A1	H142	12/M	2011/10/04	GII.P21/GII.3	G4P[8]	‒	AdV-41	‒	‒	KU362769^d^, KU362775^a^, KU362781^b^, KU362788^c^
	A1	H144	2.5/F	2011/09/30	‒	‒	‒	**+**	‒	‒	KU362770^d^, KU362776^a^, KU362782^b^, KU362789^c^
	A1	H159	6/F	2011/10/20	GII.P21/GII.3	‒	‒	AdV-6	‒	‒	KU362771^d^, KU362777^a^, KU362783^b^, KU362790^c^
	A1	H169	39/M	2012/02/18	‒	G3P[8]	‒	‒	‒	‒	KU362791^c^
	A1	H214	7/M	2011/11/15	‒	‒	HAstV-5	‒	‒	‒	KU362772^d^, KU362778^a^, KU362792^c^
**BuV**	1	H040	2.5/M	2011/01/30	GII	**+**	‒	AdV-6	‒	‒	KU362760^a^, KU362762^d^
	1	H232	39/M	2012/03/05	‒	GxP[8]	‒	AdV-2	‒	‒	KU362761^a^, KU362763^d^

CosV: cosavirus; SalV: salivirus; BuV: bufavirus; NoV: norovirus; RVA: rotavirus; AstV: astrovirus; AdV: adenovirus; SaV: sapovirus; AiV: Aichi virus

* Genotypes are shown when available; ‒: negative; +: positive; M: male; F: female

^a^ Polymerase region

^b^ Helicase region

^c^ 5’UTR region

^d^ VP region

**Fig 2:**
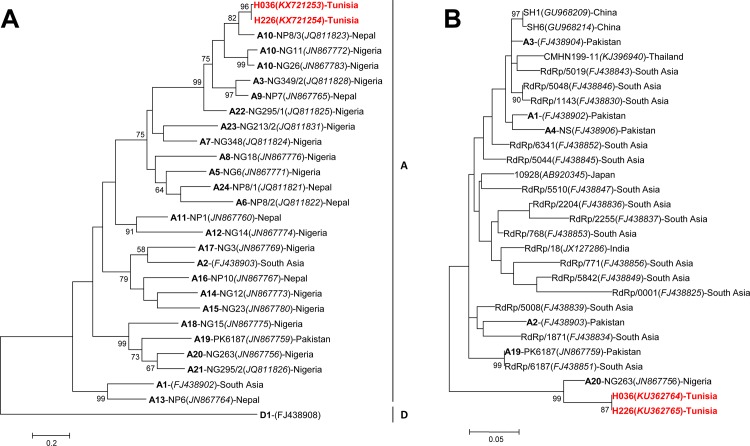
Phylogenetic trees of cosaviruses detected in diarrheal Tunisian children. **A.** VP1 region of cosavirus (904 nt); **B.** 3Dpol region of cosavirus (400 nt). Phylogenetic trees were inferred using the Maximum Likelihood method based on the Tamura-3-parameter nucleotide substitution model with a discrete gamma distribution. Bootstraps values were calculated from 1000 replicates. Strains of this study are shown in red. Genotypes are shown in bold.

Our data showed that the detection rate of CosV in diarrheal children (1%) was lower than observed in China or Brazil, where they were reported in diarrheal children in 2.8% and 3.6% of patients **[33, 34]**, respectively. CosV has been previously reported in up to 33.0% of healthy Tunisian subjects or suffering from non-polio acute flaccid paralysis, most of them being children <6 years old **[35]**. However, the difference in detection rates is due to different natures of the two cohorts. According to phylogenetic analysis of their VP1 regions, the two CosV strains, H036 and H226, were closely related to the genotype A10. Indeed, they shared 87% and 88% of their nucleotide sequences, respectively, but 98% of their amino acids (aa) sequences with the Nepalese NP8/3 CosV strain (JQ811823) **(Fig 2A)**. Since the 3Dpol sequence of the NP8/3 strain is not known and the length of the sequences is short, their 3Dpol regions were closely related to the Nigerian NG263 CosV strain (JN867756), which belongs to the genotype A20, with 93% and 94% of nucleotide sequence homology, respectively **(Fig 2B)**. Of note, the VP1 sequences of our strains shared merely 56% of their aa sequences with the NG263 strain.

**Fig 3:**
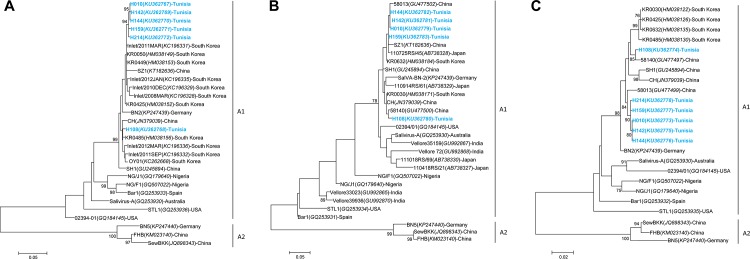
Phylogenetic trees of saliviruses detected in diarrheal Tunisian children. **A.** VP0 region of salivirus (815 nt); **B.** 2CHel region of salivirus (275 nt); **C.** 3Dpol region of (686 nt); Phylogenetic trees were inferred using the Maximum Likelihood method based on the Tamura-3-parameter nucleotide substitution model with a discrete gamma distribution. Bootstraps values were calculated from 1000 replicates. Strains of this study are shown in blue. Genotypes are shown in bold.

In this study, the detection rate of SalV infections accounted for 3.5% of the Tunisian cases, which is close to the 4.2% observed in Chinese children but much less than the 8.8% of cases in South Korea **[12, 13]**. Although they are globally widespread, SalV seem to circulate more in Asia that in North Africa. Interestingly, the detection rate of SalV in this study was similar to the detection rate of AiV (3.6%) in Tunisian children **[36]**. Since *Salivirus* is a genus phylogenetically close to *Kobuvirus*, both viruses might share some epidemiology characteristics that remain to be defined. Phylogenetic analysis of VP0, 2Chel and 3Dpol regions of 6 of the 7 detected SalV also showed that they belonged to the cluster A1 and were all closely related with various Asian strains, especially from South Korea **(Fig 3)**. Further analysis also showed that the nt and aa sequences of the Tunisian clustering strains (i.e. strains H010, H142, H144, H159 and H214) had 99% of homology with Hungarian strains detected 1 year later in newborns suffering from AGE **[5]** (data not show).

**Fig 4:**
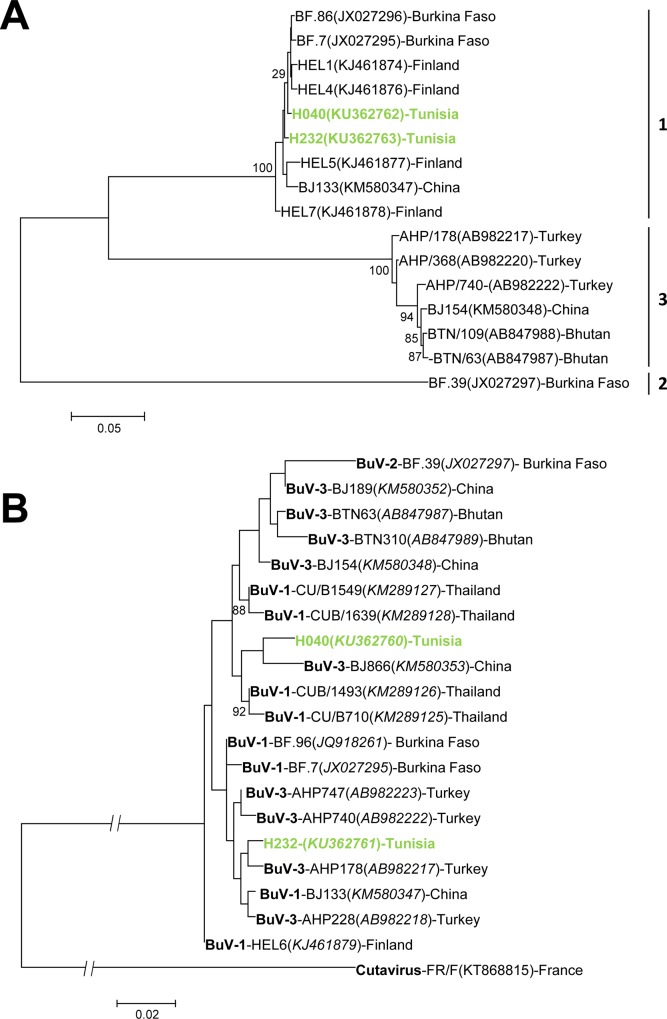
Phylogenetic trees of bufaviruses detected in diarrheal Tunisian children. **A.** VP2 region of bufavirus (1710 nt); **B.** NS1 region of bufavirus (441 nt) Phylogenetic trees were inferred using the Maximum Likelihood method based on the Tamura-3-parameter nucleotide substitution model with a discrete gamma distribution. Bootstraps values were calculated from 1000 replicates. Strains of this study are shown in green. Genotypes are shown in bold.

With a low detection rate (1%), BuV were only found occasionally in Tunisian stools. These findings are similar to those observed in children from Asia, Europe or Africa where detection rates range from 0.5% to 4.0% in patients of all ages **[6–10]**. Given that this present study was focused only on young children, our results suggest that BuV detection rate is the similar in young children as the rest of the Tunisian population. Complete VP2 sequences of H040 and H232 BuV strains showed that both strains belonged to genotype 1 and shared 99% of their sequence with published West-African and Finnish strains **(Fig 4A)**. The 2 BuV strains shared 99% of their VP2 aa sequences (7/569 aa substitutions). Although NS1 sequences appeared more closely related to strains from genotype 3 **(Fig 4B)**, aa sequence analysis of BuV polymerase showed small divergence between genotypes. Serological studies will be needed to get a better picture of BuV circulation in Tunisian population.

## Conclusion

Although these new viruses are suspected to be responsible for AGE in children, our data showed that this association was uncertain since all infected children also presented infections with several enteric viruses. Nevertheless, these multiple infections exemplified the threat that enteric viruses pose in terms of public health within communities in North Africa and generally in developing countries. Therefore, further studies with large cohorts of healthy and diarrheal children will be needed to evaluate their clinical role in AGE.

## Supporting Information

S1 File GenBank Accession Numbers. (DOCX)

S1 Table Oligonucleotides used for detection and genotyping of classic enteric viruses in this study. (PDF)
