# Supplementary material for: Cosavirus, Salivirus and Bufavirus in Diarrheal Tunisian Infants
Source: PLoS One. 2016 Sep 15;11(9):e0162255. doi: 10.1371/journal.pone.0162255 (PMC5025138; doi:10.1371/journal.pone.0162255)
Supplement: S1 File — (DOCX) [file pone.0162255.s001.docx]

**GenBank accession numbers:**

**Cosavirus:** KU362764: CosV/Human-wt/TUN/H036/2011/CosV-A10/3Dpol; KU362765: CosV/Human-wt/TUN/H226/2012/CosV-A10/3Dpol; KU362766: CosV/Human-wt/TUN/H036/2011/CosV-A10/2Chel; KU362784: CosV/Human-wt/TUN/H036/2011/CosV-A10/5’UTR; KU362785: CosV/Human-wt/TUN/H226/2012/CosV-A10/5’UTR; KX721253: CosV/Human-wt/TUN/H036/2011/CosV-A10/VP1; KX721254: CosV/Human-wt/TUN/H226/2012/CosV-A10/VP1.

**Salivirus:** KU362767: SalV/Human-wt/TUN/H010/2011/SalV-A/VP; KU362768: SalV/Human-wt/TUN/H108/2011/SalV-A/VP; KU362769: SalV/Human-wt/TUN/H142/2011/SalV-A/VP; KU362770: SalV/Human-wt/TUN/H144/2011/SalV-A/VP; KU362771: SalV/Human-wt/TUN/H159/2011/SalV-A/VP; KU362772: SalV/Human-wt/TUN/H214/2011/SalV-A/VP; KU362773: SalV/Human-wt/TUN/H010/2011/SalV-A/3Dpol; KU362774: SalV/Human-wt/TUN/H108/2011/SalV-A/3Dpol; KU362775: SalV/Human-wt/TUN/H142/2011/SalV-A/3Dpol; KU362776: SalV/Human-wt/TUN/H144/2011/SalV-A/3Dpol; KU362777: SalV/Human-wt/TUN/H159/2011/SalV-A/3Dpol; KU362778: SalV/Human-wt/TUN/H214/2011/SalV-A/3Dpol; KU362779: SalV/Human-wt/TUN/H010/2011/SalV-A/2Chel; KU362780: SalV/Human-wt/TUN/H108/2011/SalV-A/2Chel; KU362781: SalV/Human-wt/TUN/H142/2011/SalV-A/2Chel; KU362782: SalV/Human-wt/TUN/H144/2011/SalV-A/2Chel; KU362783: SalV/Human-wt/TUN/H159/2011/SalV-A/2Chel; KU362786: SalV/Human-wt/TUN/H010/2011/SalV-A/5’UTR; KU362787: SalV/Human-wt/TUN/H108/2011/SalV-A/5’UTR; KU362788: SalV/Human-wt/TUN/H142/2011/SalV-A/5’UTR; KU362789: SalV/Human-wt/TUN/H144/2011/SalV-A/5’UTR; KU362790: SalV/Human-wt/TUN/H159/2011/SalV-A/5’UTR; KU362791: SalV/Human-wt/TUN/H169/2012/SalV-A/5’UTR; KU362792: SalV/Human-wt/TUN/H214/2011/SalV-A/5’UTR.

**Bufavirus:** KU362760: BuV/Human-wt/TUN/H040/2011/BuV-1/NS1; KU362761: BuV/Human-wt/TUN/H0232/2012/BuV-1/NS1; KU362762: BuV/Human-wt/TUN/H040/2011/BuV-1/VP2; KU362763: BuV/Human-wt/TUN/H232/2012/BuV-1/VP2.
