# Supplementary material for: Cosavirus, Salivirus and Bufavirus in Diarrheal Tunisian Infants
Source: PLoS One. 2016 Sep 15;11(9):e0162255. doi: 10.1371/journal.pone.0162255 (PMC5025138; doi:10.1371/journal.pone.0162255)
Supplement: S1 Table — (PDF) [file pone.0162255.s002.pdf]

S1 Table. Oligonucleotides used for detection and genotyping of classic enteric viruses in this study

Virus	Method	Primer/probe	Nucleotide Sequence	Kit	Reference
Norovirus GI	RT-qPCR (screening)	JJV1NF	CCA TGT TCC GTT GGA TGC	TaqMan® fast Virus 1-Step (Applied Biosystems)	Lyman *et al*, 2009 [17]
		JJV1R	TCC TTA GAC GCC ATC ATC AT		
		RING-1b *	AGA TCG CGG TCT CCT GTC CA		
	RT-PCR (typing)	G1SKF	CTG CCC GAA TTY GTA AAT GA	One-Step® RT-PCR (Qiagen)	Kojima *et al*, 2002 [18]
		G1SKR	CCA ACC CAR CCA TTR TAC A		
Norovirus GII	RT-qPCR (screening)	QNIF2d	ATG TTC AGR TGG ATG AGR TTC TCW GA	TaqMan® fast Virus 1-Step (Applied Biosystems)	Da Silva *et al*, 2007 [19]
		COG2R	TCG ACG CCA TCT TCA TTC ACA AGA TCG		
		QNIFs *	AGC ACG TGG GAG GGG ATC		
	RT-PCR (typing)	G2SKF	CNT GGG AGG GCG ATC GCA A	One-Step® RT-PCR (Qiagen)	Kojima *et al*, 2002 [18]
		G2SKR	CCR CCN GCA TRH CCR TTR TAC AT		
Rotavirus	RT-qPCR (screening)	VP2-F1	TCT GCA GAC AGT TGA ACC TAT TAA	TaqMan® fast Virus 1-Step (Applied Biosystems)	Gutierrez-Aguirre *et al*, 2008 [20]
		VP2-F2	CAG ACA CGG TTG AAC CCA TTA A		
		VP2-F3	TCG GCT GAT ACA GTA GAA CCT ATA AAT G		
		VP2-F4	TGT CAG CTG ATA CAG TAG AAC CTA TAA ATG		
		VP2-F5	TCA GCT GAC ACA GTA GAA CCT ATA AAT G		
		VP2-R1	GTT GGC GTT TAC AGT TCG TTC AT		
		VP2-R2	GTT GGC GTC TAC AAT TCG TTC AT		
		VP2-P *	ATG CGC ATR TTR TCA AAH GCA A		
	RT-PCR (typing)	VP7-F	ATG TAT GGT ATT GAA TAT ACC AC	One-Step® RT-PCR (Qiagen)	Iturriza-Gómara *et al*, 2001 [21]
		VP7-R	AAC TTG CCA CCA TTT TTT CC		
		G1	CAA GTA CTC AAA TCA ATG ATG G	AmpliTaq® DNA Polymerase (Applied Biosystems)	EuroRotaNet, 2009 [22]
		G2	CAA TGA TAT TAA CAC ATT TTC TGT G		
		G3	ACG AAC TCA ACA CGA GAG G		
		G4	CGT TTC TGG TGA GGA GTT G		
		G8	TTR TCG CAC CAT TTG TGA AAT		
		G9	CTT GAT GTG ACT AYA AAT AC		
		G10	ATG TCA GAC TAC ARA TAC TGG		
		G12	GGT TAT GTA ATC CGA TGG ACG		
		VP7-RINT	ANA YNG ANC CWG TYG GCC A		
		VP4-F	TAT GCT CCA GTN AAT TGG	One-Step® RT-PCR (Qiagen)	Gentsch *et al*, 1992 [23]
		VP4-R	ATT GCA TTT CTT TCC ATA ATG		
		2T-1	CTA TTG TTA GAG GTT AGA GTC	AmpliTaq® DNA Polymerase	EuroRotaNet, 2009 [22]

		3T-1<br>1T-1D<br>4T-1<br>5T-1<br>P[11]	TGT TGA TTA GTT GGA TTC AA<br>TCT ACT GGR TTR ACN TGC<br>TGA GAC ATG CAA TTG GAC<br>ATC ATA GTT AGT AGT CGG<br>GTA AAC ATC CAG AAT GTG	(Applied Biosystems)	
Astrovirus	RT-qPCR (screening)	AV1<br>AV2<br>AVs *	CCG AGT AGG ATC GAG GGT<br>GCT TCT GAT TAA ATC AAT TTT AA<br>CTT TTC TGT CTC TGT TTA GAT TAT TTT AAT CAC C	TaqMan® fast Virus 1-Step (Applied Biosystems)	Le Cann *et al*, 2004 **[24]**
	RT-PCR (typing)	MON244<br>MON245	GGT GTC ACA GGA CCA AAA CC<br>TTA GTG AGC CAC CAG CCATC	One-Step® RT-PCR (Qiagen)	Noel *et al*, 1995 **[25]**
Sapovirus	RT-qPCR (screening)	SaV124F<br>SaV1F<br>SaV1245R<br>SaV124TP *	GAY CAS GCT CTC GCY ACC TAC<br>TTG GCC CTC GCC ACC TAC<br>CCC TCC ATY TCA AAC ACT A<br>CCR CCT ATR AAC CA	TaqMan® fast Virus 1-Step (Applied Biosystems)	Oka *et al*, 2006 **[26]**
	RT-PCR (typing)	SR80<br>NV110	TGG GAT TCT ACA CAA AAC CC<br>ACD ATY TCA TCA TCA CCA TA	One-Step® RT-PCR (Qiagen)	Noel *et al*, 1997 **[27]**<br>Le Guyader *et al*, 1996 **[28]**
Adenovirus	qPCR (screening)	-	-	Adenovirus R-Gene® (Argene)	-
	PCR (typing)	Hex1DEG<br>Hex2DEG	GCC SCA RTG GKC WTA CAT GCA CAT C<br>CAG CAC SCC ICG RAT GTC AAA	AmpliTaq® DNA Polymerase (Applied Biosystems)	Allard *et al*, 2001 **[29]**
Aichi virus	RT-PCR (screening and typing)	6261<br>6779	ACA CTC CCA CCT CCC GCC AGT A<br>GGA AGA GCT GGG TGT CAA GA	One-Step® RT-PCR (Qiagen)	Yamashita *et al*, 2000 **[30]**

* Probes
